# Relying on injection drug users to prevent HIV in Ukraine - follow-up results of peer-driven interventions

**DOI:** 10.1186/1742-4690-9-S1-P104

**Published:** 2012-05-25

**Authors:** Oleksandra Datsenko, Pavlo Smyrnov, Robert Broadhead

**Affiliations:** 1 International Hiv-Aids Alliance-Ukraine, Kyiv, Ukraine

## Introduction

Conventional harm reduction (HR) projects overlook IDUs’ capabilities in offering them services but no active roles to play in preventing HIV. In contrast, “peer-driven interventions” (PDIs) offer IDUs rewards to educate and recruit peers for services. All IDU-recruits receive the opportunity to play both roles.

## Methods

In 2010, the International HIV/AIDS Alliance-Ukraine implemented PDIs in 12 Ukrainian cities that relied entirely on IDUs to access and teach IDUs who either had: (a.) never received HR services, or (b.) former PDI/HR-respondents eligible for a one-year follow-up (FU) intervention. IDU-recruiters were trained to administer two completely different bodies of prevention information: one to new recruits, the other to FU-recruits. Recruits from both groups were administered an 8-point knowledge test (KT) at their appointment that measured how well their recruiters educated them.

## Results

In 6 months of operation, the 12 PDIs recruited 8,115 IDUs:

2,782 (34%) new recruits

5,333 (66%) PDI-FU-recruits

- New recruits: 87% scored 7 or higher on the first KT

- FU-recruits were administered both KTs at the follow-up appointment.

86.4% scored 7 or higher on the 1st KT

89.6% scored 7 or higher on the 2nd KT

(One year earlier, only 81.5% of the FU-group scored 7 or higher, which underscores how the PDI’s repetitive features improved respondents’ retention rate.)

## Conclusion

The PDIs documented that IDUs can play active roles in preventing HIV by recruiting IDU-peers who have never received HR services, or IDUs eligible for FU intervention. They are also able to deliver two entirely different bodies of prevention information. Compared to HR projects that relied on traditional staffs of salaried outreach workers, the PDI proved to be far more powerful and cost-effective model. Alliance-Ukraine now plans to broaden its investment in PDIs even more heavily by further expanding projects targeting IDUs, but also female sex workers and homeless/runaway street children.

